# Unveiling Topics and Emotions in Arabic Tweets Surrounding the COVID-19 Pandemic: Topic Modeling and Sentiment Analysis Approach

**DOI:** 10.2196/53434

**Published:** 2025-02-10

**Authors:** Farah Alshanik, Rawand Khasawneh, Alaa Dalky, Ethar Qawasmeh

**Affiliations:** 1 Department of Computer Science Faculty of Computer and Information Technology Jordan University of Science and Technology Irbid Jordan; 2 Department of Clinical Pharmacy Faculty of Pharmacy Jordan University of Science and Technology Irbid Jordan; 3 Department of Health Management and Policy Faculty of Medicine Jordan University of Science and Technology Irbid Jordan; 4 Department of Computer Science Faculty of Computer Science and Engineering The Ohio State University Columbus, OH United States

**Keywords:** topic modeling, sentiment analysis, COVID-19, social media, Twitter, public discussion

## Abstract

**Background:**

The worldwide effects of the COVID-19 pandemic have been profound, and the Arab world has not been exempt from its wide-ranging consequences. Within this context, social media platforms such as Twitter have become essential for sharing information and expressing public opinions during this global crisis. Careful investigation of Arabic tweets related to COVID-19 can provide invaluable insights into the common topics and underlying sentiments that shape discussions about the COVID-19 pandemic.

**Objective:**

This study aimed to understand the concerns and feelings of Twitter users in Arabic-speaking countries about the COVID-19 pandemic. This was accomplished through analyzing the themes and sentiments that were expressed in Arabic tweets about the COVID-19 pandemic.

**Methods:**

In this study, 1 million Arabic tweets about COVID-19 posted between March 1 and March 31, 2020, were analyzed. Machine learning techniques, such as topic modeling and sentiment analysis, were applied to understand the main topics and emotions that were expressed in these tweets.

**Results:**

The analysis of Arabic tweets revealed several prominent topics related to COVID-19. The analysis identified and grouped 16 different conversation topics that were organized into eight themes: (1) preventive measures and safety, (2) medical and health care aspects, (3) government and social measures, (4) impact and numbers, (5) vaccine development and research, (6) COVID-19 and religious practices, (7) global impact of COVID-19 on sports and countries, and (8) COVID-19 and national efforts. Across all the topics identified, the prevailing sentiments regarding the spread of COVID-19 were primarily centered around anger, followed by disgust, joy, and anticipation. Notably, when conversations revolved around new COVID-19 cases and fatalities, public tweets revealed a notably heightened sense of anger in comparison to other subjects.

**Conclusions:**

The study offers valuable insights into the topics and emotions expressed in Arabic tweets related to COVID-19. It demonstrates the significance of social media platforms, particularly Twitter, in capturing the Arabic-speaking community’s concerns and sentiments during the COVID-19 pandemic. The findings contribute to a deeper understanding of the prevailing discourse, enabling stakeholders to tailor effective communication strategies and address specific public concerns. This study underscores the importance of monitoring social media conversations in Arabic to support public health efforts and crisis management during the COVID-19 pandemic.

## Introduction

### Background

Throughout history, humanity has faced numerous outbreaks of infectious diseases that have resulted in significant loss of life and economic impact. Toward the end of 2019, the World Health Organization reported a series of pneumonia cases in Wuhan, which were later identified as COVID-19. As a novel infectious disease transmitted through respiratory droplets and contact, COVID-19 quickly spread across the globe, leading to an unprecedented impact on global public health, businesses, and economies. As of February 7, 2023, there have been >676 million confirmed cases and 500,000 reported deaths in >200 countries [1]. Social media platforms, particularly Twitter, have emerged as valuable sources of information for understanding and predicting disease outbreaks. Text mining techniques allow for the extraction of relevant health information from user-generated content on social media platforms. Twitter, in particular, provides researchers with vast amounts of real-time data, enabling early response strategies and enhancing situational awareness. Analyzing Twitter data has become a crucial area of focus in medical informatics research [2,3].

COVID-19 emerged as a prominent and sustained topic on Twitter starting from January 2020, and its discussion has persisted uninterrupted up to the present day [4]. With quarantine measures implemented worldwide, individuals increasingly relied on social media to access news and express their opinions. Twitter data offer valuable insights into public discussions, sentiments, and real-time updates during global pandemics [2,5]. Using Twitter as a data source enables infodemiology studies, providing health authorities with opinions and concerns to inform their responses [6].

Since the outset of the COVID-19 outbreak, an escalating number of studies have been harnessing Twitter data to delve into the public’s reactions and discussions surrounding the COVID-19 pandemic. In their respective studies, researchers used distinct methodologies to explore COVID-19–related discussions and sentiments. For instance, Xue et al [4,7] used latent Dirichlet allocation (LDA) for topic identification. Similarly, a study by Alharbi and Alkhateeb [8] investigated the sentiment of the Arabic public on Twitter, using natural language processing (NLP) and machine learning techniques, finding that the long short-term memory model outperformed the naive Bayes model with an accuracy rate of 99% [8]. Another study focused on Arabic sentiment analysis for vaccine-related COVID-19 tweets, introducing the first and largest human-annotated dataset in Arabic for this purpose; it used advanced models such as the stacked gated recurrent unit and AraBERT, achieving a 7% accuracy enhancement [9]. During the COVID-19 pandemic, a separate study analyzed online learning–related tweets in Arabic, using various classification algorithms and achieving a maximum accuracy of approximately 89.6% using the Support Vector Machine classifier to analyze public perceptions of the coronavirus [10]. In addition, research conducted in Saudi Arabia showed a significant increase in negative sentiments during the COVID-19 pandemic, with deep learning algorithms achieving high accuracy rates [11]. Other studies explored sentiment differences between countries and in response to events, using topic modeling and sentiment analysis to reveal previously unreported patterns [12]. Furthermore, a study from Morocco compared different machine learning algorithms for tweet classification, finding logistic regression to yield the best sentiment predictions [13].

Recent advancements in NLP have shown significant potential in transforming various aspects of health care, including clinical decision support, patient management, and automated analysis of health records. Recent studies, such as the one by Tamang et al [14], highlight the use of NLP for optimizing patient outcome predictions and identifying disease patterns through electronic health record data. Similarly, a study by Elbattah et al [15] explores the role of NLP in extracting actionable insights from unstructured medical texts, further underscoring the growing relevance of NLP in enhancing the health care decision-making processes.

COVID-19 remains a scientifically and medically novel disease that requires in-depth and consistent research. Leveraging social media data, particularly from platforms such as Twitter, is essential for syndromic surveillance and understanding public health–related concerns. Twitter, as a prominent communication modality during disease outbreaks, offers valuable insights into public awareness and provides real-time reflections of public sentiment. Despite extensive research on COVID-19, limited studies have used social media data, specifically Twitter, to address conclusive themes and sentiment analysis in Arab regions during the early stages of the COVID-19 pandemic.

While numerous studies have investigated similar themes in different languages and contexts, there remains a notable gap in the analysis of Arabic tweets [16-22]. The Arabic-speaking population plays a significant role in the global discourse on COVID-19, and their perspectives and sentiments warrant dedicated exploration. Building on previous research, and to bridge this gap, our study used a combination of topic modeling techniques, specifically LDA, and sentiment analysis methods to uncover the predominant topics of discussion and the prevailing emotional tones within this corpus.

### This Study

This study aims to analyze Twitter posts during the early stages of the COVID-19 pandemic in Arab regions to provide valuable insights into public sentiment, concerns, and awareness regarding COVID-19 in Arab communities. To achieve this, >1 million tweets posted between March 1 and March 31, 2020, were collected and analyzed. Through this analysis, we hope to assist policy makers in making informed decisions, enhancing public health communication, and implementing effective interventions to mitigate the impact of future outbreaks.

Although this study was conducted during the COVID-19 pandemic, its scope extends beyond the immediate implications of the COVID-19 pandemic. The primary goal of this research is to enhance health care planning and resource allocation in Jordan, which remains a critical issue regardless of pandemic conditions. The findings are designed to inform strategies that could be beneficial in various health care scenarios, whether in routine health care management or in response to other emergent public health challenges. Therefore, the study’s relevance persists even in a postpandemic context, making it valuable for long-term health care system improvements.

## Methods

### Research Design

**Figure 1:**
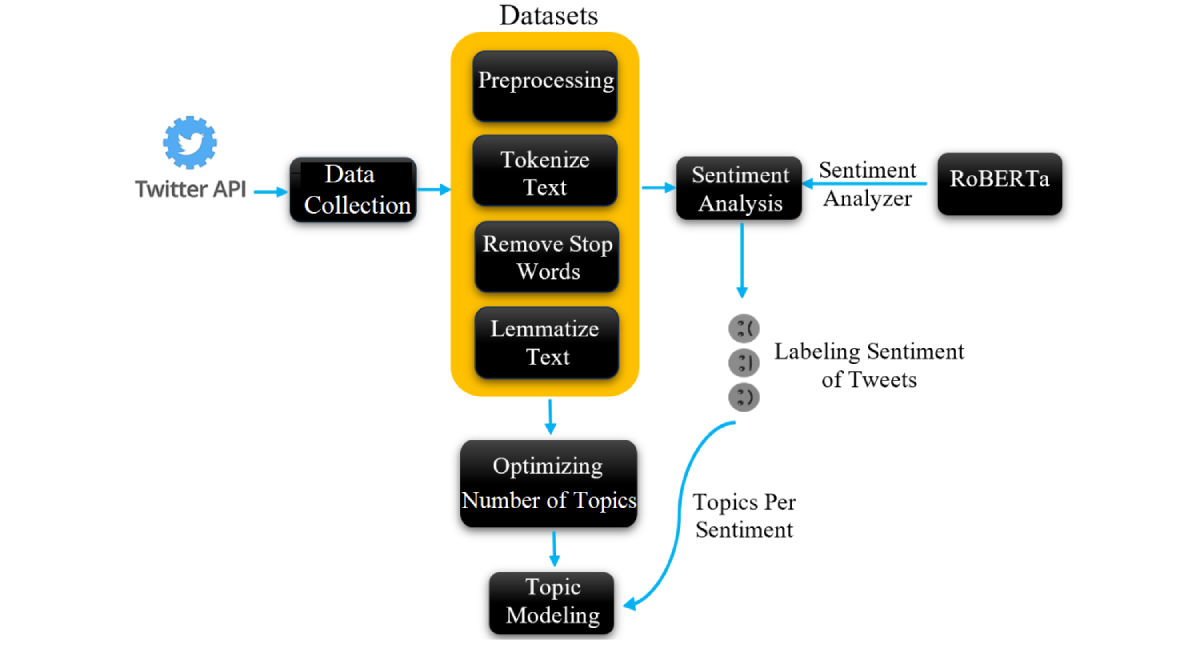
Twitter data mining pipeline. API: application programming interface.

This study uses LDA for topic modeling and a sentiment analysis emotion detection tool to uncover topics and emotions in Twitter data related to COVID-19 in the Arab region. The methodological flowchart is depicted in Figure 1. Our approach to mining Twitter data adheres to the following 4 primary steps: data collection, data preprocessing, sentiment analysis, and topic modeling. The flowchart in Figure 1 illustrates how these steps are interconnected and carried out in our data analysis pipeline. Through these methods, we aim to gain valuable insights into the topics of discussion and the emotional responses of individuals in the Arab region concerning the COVID-19 pandemic.

### Data Collection

In our research, we harnessed the GeoCoV19 dataset, a multilingual COVID-19 Twitter dataset that spans a significant period of 90 days, from February 1, 2020, to May 1, 2020. This extensive dataset comprises hundreds of millions of tweets and is enriched with a diverse set of multilingual hashtags and keywords to ensure its comprehensiveness [23]. The dataset primarily provides tweet IDs, which presented us with the task of retrieving the actual tweet text associated with these IDs. To accomplish this, we made effective use of the Twarc application programming interface (API), a robust and efficient tool explicitly designed for this purpose [24]. The Twarc library was chosen due to its robustness in handling large-scale data collection, effective management of Twitter’s API rate limits, seamless integration with existing data pipelines, and support for extended tweet metadata, making it an ideal tool for ensuring the integrity and completeness of the dataset required for this study. The Twarc API streamlined the process of collecting tweet texts corresponding to the tweet IDs provided. As we gathered all the tweets, we applied a language filter to focus exclusively on Arabic tweets. This selective filtering step was crucial for tailoring the dataset to our specific analysis, concentrating on tweets in the Arabic language.

### Data Preprocessing

Data preprocessing plays a pivotal role in text mining, and it serves as a fundamental step in this domain. The purpose of this preprocessing is 2-fold: it optimizes the efficiency of prediction algorithms by eliminating potentially detrimental words, and it conserves storage space, contributing to improved computational performance [25]. In our analysis, we worked with Arabic text data, which requires thorough preprocessing to filter out any noise or irrelevant elements. The initial raw Arabic text underwent a series of transformations as part of this preprocessing effort. These transformations involved tokenization and the removal of various elements such as white spaces, punctuation marks, special characters, emojis, and URLs. To accomplish this, we used a set of established methods for Arabic text preprocessing, including the use of Farasa [26]. Farasa proved invaluable in normalizing Arabic characters, stripping away diacritics, erasing punctuation marks, and eliminating repetitive characters, collectively enhancing the quality and relevance of the text data for our analysis.

### Sentiment Analysis

#### Overview

To classify the primary sentiments expressed in Twitter messages, such as fear and joy, we used sentiment analysis, an NLP technique [27]. Our approach involved deploying the RoBERTa-base model, meticulously trained on a vast corpus of approximately 58 million tweets and further fine-tuned for precise emotion recognition leveraging the TweetEval benchmark [28]. This specific model, known as Twitter-RoBERTa-Base-Emotion [29], has been purposefully tailored for the nuanced task of emotion recognition within Twitter text data. It adeptly classifies text into various emotion categories, including joy, sadness, anger, fear, surprise, disgust, anticipation, and trust. Our sentiment analysis process unfolded in a sequence of four distinct steps, described in the following sections.

#### Step 1: Translation to English

As a reliable Arabic emotion detection API was not readily available, we initiated the process by translating Arabic tweets to English. To accomplish this, we leveraged the Google Translation API. We established an account and procured the necessary translation service. It is worth noting that the cost associated with using the Google Translation API amounts to US $20 per 1 million characters. Given that we were dealing with a substantial volume of data, encompassing 5.1 million Arabic tweets with a staggering 970,801,329 characters, the estimated cost tallied up to US $19,420. Consequently, we opted to translate 1 entire month of tweets. March was selected as the ideal candidate for translation, primarily due to its status as the month with the highest tweet volume. In addition, March witnessed several pivotal events, including Trump’s declaration of COVID-19 as a national emergency, the implementation of travel bans on non-US citizens traveling from Europe, and the World Health Organization’s formal declaration of the coronavirus as a global pandemic. To verify the quality of the translations, a sample of 5000 tweets was randomly selected and evaluated both before and after translation. Bilingual experts reviewed these tweets, comparing the original Arabic content with the translated English text. This review process focused on ensuring that the translations accurately conveyed the original meaning, context, and sentiment. On the basis of their feedback, we confirmed that the translations were of high quality, making them suitable for further analysis.

#### Step 2: English Text Preprocessing

Once the translation was complete, we embarked on preprocessing the English text. This entailed removing common stop words such as “and,” “the,” and “to.”

#### Step 3: Stemming

To further refine the text data, we applied a stemming process, which involves eliminating predefined prefixes and suffixes. This step aids in reducing words to their root form. For instance, it transforms “running” into “run” through stemming.

#### Step 4: Emotion Determination

The final step involved determining the emotion expressed in the tweets using Twitter-RoBERTa-Base-Emotion.

Table 1 illustrates the distribution of emotions across the analyzed tweets, providing valuable insights into the prevailing sentiments during the specified time frame.

**Table 1:** Number of tweets per emotion.

Emotion	Tweets, n
Anger	182,105
Disgust	150,022
Joy	141,446
Anticipation	60,449
Sadness	44,591
Surprise	30,666
Fear	28,439

### Topic Modeling Using LDA

In our analysis, we harnessed the power of LDA as a formidable tool for uncovering latent topics within our extensive dataset. LDA, a generative probabilistic model, proves exceptionally useful for extracting these hidden themes from a vast collection of documents. Its underlying mechanism involves representing documents as random combinations of latent topics and characterizing each topic as a distribution of words [30]. This framework of the LDA model adheres to a 3-level Bayesian approach to effectively capture the generative process. However, before delving into the application of LDA or any other probabilistic topic modeling techniques, a critical step is to determine and define the number of topics often denoted as “k” [31]. This crucial decision significantly impacts the outcomes of the topic modeling process.

### Qualitative Analysis

To strengthen the reliability of our findings obtained through the LDA model, we integrated a qualitative method focused on gaining a more profound insight into the identified themes. In particular, we followed the established 6-step thematic analysis framework outlined by Braun and Clarke [32] and successfully used by Xue et al [33]. This framework includes the following steps: (1) familiarizing ourselves with the keyword data and reviewing the most representative tweets for each topic, (2) generating initial codes to summarize key themes, (3) searching for thematic patterns by grouping similar topics, (4) reviewing and refining these potential themes to ensure coherence and consistency, (5) defining and naming themes based on their overall significance and contribution to the research question, and (6) reporting and documenting the final themes. This process was iterative and reflexive, involving multiple rounds of discussion and reassessment. Two researchers with extensive experience in social media analysis and public health independently reviewed and documented the initial codes. These codes were then examined by 2 additional researchers to refine the themes, ensuring that they accurately captured the essence of the topics.

### Ethical Considerations

This study analyzed publicly available data collected from Twitter. The dataset consisted of tweet IDs, and no personally identifiable information was included in the analysis. All tweet texts were retrieved in compliance with Twitter’s terms of service. Ethics approval was not sought, as the study used publicly accessible data, ensuring that no identifiable personal information was involved. To maintain the highest ethical standards, all results are presented in aggregate, guaranteeing the anonymity and privacy of individuals represented in the dataset.

## Results

### Descriptive Results

A total of 637,718 tweets were included in the final dataset after processing raw data. The analysis focused on identifying the most frequently tweeted bigrams (pairs of words) related to COVID-19. Bigrams are 2 consecutive words, regardless of their grammar structure or semantic meaning. They may not be self-explanatory, as in the case of the bigram “social distancing,” which does not convey the meaning of either word on its own. Such an approach was adopted by Xue et al [4], and it was proved that bigrams can be a useful way to identify the most prominent topics and themes in Twitter conversations. The identified bigrams included pairs of words such as “virus corona,” “stay home,” “home order,” “travel curfew,” “new coronavirus,” “spread virus,” “home quarantine,” “health quarantine,” “coronavirus pandemic,” “new infected,” and “new case.” Among the popular unigrams were words such as “coronavirus,” “virus,” “home,” “new,” “health,” “world,” “visit,” “pandemic,” “stay,” “case,” “quarantine,” and “curfew.” Most common unigrams and bigrams related to COVID-19, and pertinent details are listed in Table 2 (original Arabic tweets are provided in Multimedia Appendix 1).

**Table 2:** Top 50 unigrams and bigrams and their distributions.

			Values (%)
**Top 50 unigrams**			
	Coronavirus	6.558451	
	Virus	2.350919	
	Home	0.921041	
	New	0.857981	
	Health	0.614924	
	Kuwait	0.576566	
	Condition	0.551307	
	Saudi Arabia	0.503562	
	World	0.491143	
	Country	0.487031	
	Visit	0.392251	
	Pandemic	0.391468	
	Curfew	0.359459	
	Stay	0.359077	
	Country	0.352204	
	Spread	0.34872	
	Infected	0.340486	
	Quarantine	0.339662	
	Case	0.335292	
	Disease	0.331376	
	Infected	0.328934	
	Urgent	0.314949	
	Egypt	0.313753	
	Virus	0.288958	
	People	0.272675	
	Minister	0.263771	
	People	0.257506	
	Health	0.244108	
	China	0.243201	
	Good	0.241965	
	Travel	0.241181	
	Citizen	0.239945	
	COVID	0.238966	
	King	0.238255	
	New	0.220993	
	Procedure	0.213274	
	Lebanon	0.211883	
	Wanted	0.209183	
	Confrontation	0.205782	
	Education	0.205174	
	In	0.198331	
	Infection	0.193302	
	Thanks	0.187623	
	Announced	0.186263	
	Prevention	0.185222	
	Nation	0.184861	
	Iran	0.180255	
	House	0.178111	
	Italy	0.174504	
	In house	0.172979	
**Top 50 bigrams**			
	Virus, coronavirus	2.029932	
	Coronavirus, new	0.526419	
	Stay home	0.325347	
	Coronavirus, coronavirus	0.302665	
	Visit, health	0.263658	
	Virus, coronavirus	0.19593	
	Coronavirus, Kuwait	0.194992	
	Coronavirus, new	0.192446	
	Curfew, travel	0.18009	
	Spread, virus	0.155542	
	Coronavirus, virus	0.146133	
	Quarantine, home	0.138868	
	Quarantine, health	0.123512	
	New, virus	0.122492	
	Coronavirus, Lebanon	0.108992	
	Pandemic, coronavirus	0.108868	
	Home, coronavirus	0.107683	
	Coronavirus, Saudi Arabia	0.105704	
	Coronavirus, Egypt	0.103818	
	Infected, virus	0.102376	
	New, case	0.09342	
	Coronavirus, COVID	0.091503	
	Kuwait, coronavirus	0.089236	
	New, coronavirus	0.088587	
	Health, global	0.08464	
	Stay, home	0.083898	
	Minister, health	0.083743	
	Crisis, coronavirus	0.083589	
	Coronavirus, stay	0.076416	
	Organizer, health	0.073128	
	Confrontation, coronavirus	0.068563	
	Condition, in	0.06845	
	Saudi Arabia, coronavirus	0.064812	
	Coronavirus, wanted	0.061967	
	Coronavirus, urgent	0.060535	
	Recording, case	0.055537	
	Confrontation, virus	0.054918	
	Spread, virus	0.053424	
	Spread, coronavirus	0.053187	
	Coronavirus, curfew	0.050755	
	Curfew, curfew	0.04958	
	Procedure, precautionary	0.049426	
	United, State	0.048818	
	Staying, home	0.048519	
	Disease, coronavirus	0.047993	
	Infected, coronavirus	0.047849	
	Citizen, resident	0.047684	
	Servant, holy mosque	0.04552	
	Prevention, travel	0.045458	
	Coronavirus, visit	0.044582	

### COVID-19–Related Topics

In our study, we used the LDA technique to identify and categorize frequently co-occurring words associated with COVID-19. The LDA algorithm allowed us to manually determine the number of topics we wanted to generate. In this study, we used 2 widely recognized metrics, CaoJuan2009 and Deveaud2014, available through the R package (R Foundation for Statistical Computing), to determine the optimal number of topics for our dataset. These metrics provided a robust framework for evaluating the coherence and distinctiveness of the topics, ensuring that the final model best captured the underlying structure of the data. The CaoJuan2009 measure is minimized when the number of topics aligns with the data’s intrinsic structure, while the Deveaud2014 measure is maximized to indicate topic coherence and separation. These metrics were used to assess and validate the number of topics to ensure they reflect the data’s diversity and relevance. By leveraging these 2 complementary metrics, we ensured that the selected number of topics provided meaningful insights and reduced the risk of overfitting. The number of topics was determined when these metrics stabilized, indicating a consistent result.

**Figure 2:**
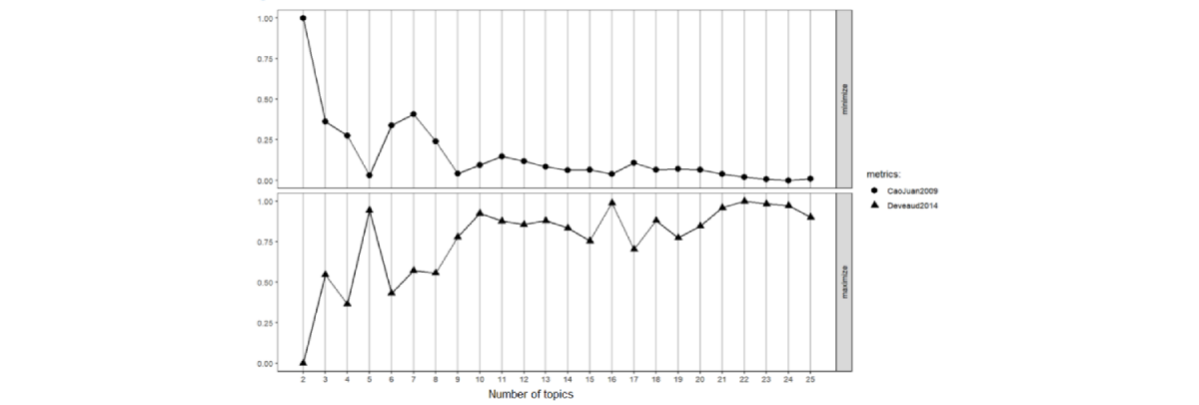
Metrics for estimating the optimal number of topics, ranging from 2 to 25 topics.

Upon evaluating the metrics, it was found that the CaoJuan2009 score converged at its minimum value with 16 topics, while the Deveaud2014 score peaked at its maximum value with the same number of topics. On the basis of this, we concluded that the optimal number of topics, denoted as “k,” is 16, as shown in Figure 2.

In addition, we calculated the topic distance and visualized the intertopic relationships using a 2D plane [34]. Each circle in the plot represents a distinct topic, ranging from topic 1 to topic k. The positioning of these circles reflects the calculated distances between topics, offering a visual representation of their relationships.

It is also worth noting that cross-validation is less commonly applied in topic modeling for several reasons. These include computational challenges associated with applying cross-validation to unsupervised models, the interpretive nature of topic models, and the emphasis on qualitative coherence over predictive performance. Most studies on LDA and related techniques do not apply cross-validation, as the focus of topic modeling is on the interpretability and coherence of the topics rather than on predictive performance. Instead, topic models are typically evaluated using internal coherence and stability measures, such as the CaoJuan2009 and Deveaud2014 metrics, which prioritize the coherence of the topics and the consistency of the results across multiple runs. This approach is consistent with what is found in most related work on LDA. For example, Blei et al [30] introduced LDA and highlighted that the evaluation of topic models is traditionally done using measures such as coherence scores.

**Figure 3:**
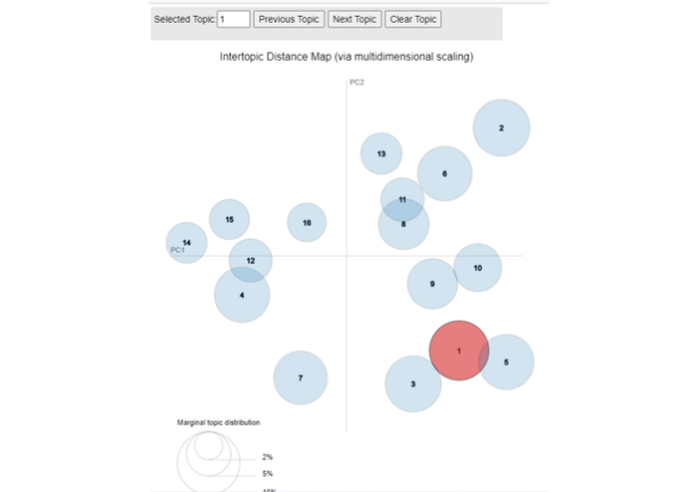
Latent Dirichlet allocation—intertopic distance.

In Table 3 (original Arabic tweets are provided in Multimedia Appendix 2), we present the findings of the 16 LDA topics, revealing the most frequently occurring words within each topic along with the percentage of tweets falling under each respective topic. Among all 16 topics, topic 5 stands out with the highest percentage (9.98%) of tweets associated with it. In topic 5, we observed a significant co-occurrence of specific words, including “coronavirus,” “increase,” “health,” “new,” “infected,” “death,” “recovery,” and “case.” This combination of words indicates an escalation in the number of COVID-19 infections, leading to unfortunate fatalities and the emergence of new cases. Moreover, the presence of the term “recovery” implies that some individuals who were previously infected are now undergoing healing and improvement. Furthermore, we calculated the topic distance and illustrated the intertopic distance [35] in a 2D plane, as depicted in Figure 3. Each circle on the plot corresponds to a topic, ranging from topic 1 to topic 16 in this study. The positions of these circles were determined based on the calculated distances between the topics. Notably, in the visualization, the circles were not overlapping, which served as a validation of the 16 topics.

**Table 3:** Topic, words, and percentage of tweets.

Topic	Words	Values (%)
0	country, corona, Kuwait, praise, protection, gratitude to god, blessing, people, protect, people or nation, state, goodness, world, Saudi Arabia, Muslim, illness, thanks, pandemic, virus, Egypt	6.31
1	corona, affliction, pandemic, goodness, virus, Muslim, mercy, supplication/prayer, new, mind, world, lift or remove, great, illness, heart, raise, evil, people, mercy, Earth	8.5
2	corona, hand, virus, mask, washing, people, new, water, sanitizer, way, discount, knowledge, world, wear, person, soap, usage, glove, mask, beautiful	4.69
3	corona, virus, illness, Iran, medical, infected, hospital, doctor, treatment, Iraq, examination or test, health, person, device, hospital, Bahrain, infected, transmission, Italy, system	7.28
4	corona, virus, Kuwait, Egypt, new, emerging, COVID, health, visited, suspension, Saudi Arabia, corona, statement, Kuwaiti, confrontation, Emirate, study, crew, state, prevention	6.09
5	corona, virus, condition, new, case, infected, health, infected, died, infection, urgent, recording, death, announced, visited, increase, recovery, recorded, total, rose	9.98
6	corona, virus, education, visited, minister, confrontation, support, private, health, student, bank, spread, sector, state, responsible, crisis, communication, community, request, home	8.16
7	corona, China, state, virus, world, pandemic, union, Italy, hate, Europe, league, America, new, spread, presented, condition, European, action, player, east	4.13
8	corona, virus, house, scene, protect, country, Algeria, Egypt, died, rest, detail, video, lead, people, young man, Morocco, new, image, wanted, film	3.83
9	house, corona, stay, curfew, quarantine, wandering, home based, virus, new, Saudi Arabia, home, Kuwait, responsible, effectiveness, roaming, health, wanted, complete, goodness, Zoom	7.18
10	corona, virus, world, Trump, Oman, new, vaccine, president, faced, America, China, treatment, wanted, news, Chinese, partnership, vaccine, COVID, American, Palestine	4.68
11	corona, virus, spread, health, state, pandemic, prevention, illness, enemy, awareness, danger, way, gathering, must, home, country, avoidance, citizen, world, prevention	5.86
12	corona, virus, spread, health, state, pandemic, prevention, illness, enemy, awareness, danger, way, gathering, must, home, country, avoidance, citizen, world, prevention	5.86
13	corona, China, state, virus, world, pandemic, union, Italy, hate, Europe, league, America, new, spread, presented, condition, European, action, player, east	4.13
14	corona, Saudi Arabia, thanks, Kuwait, king, health, protection, country, homeland, virus, citizen, people or nation, visited, effort, state, sanctuary, praise, Salman, pandemic, protect	7.35
15	corona, Lebanon, people, one, age, went out, quarantine, meant, topic, condition, house, what, virus, safety, health, Egypt, people or nation, world	4.98

### COVID-19–Related Themes

Through the process of thematic analysis, we were able to categorize the identified topics, bigrams, and representative tweet samples into distinct themes, as shown in Table 4 (original Arabic tweets are provided in Multimedia Appendix 3).

The sample tweets provided in Table 4 are excerpts taken from the original tweets. These 16 topics have been categorized into eight overarching themes, summarized below.

1. Preventive measures and safety (“public health measures”): this theme focuses on various measures to prevent the spread of COVID-19, such as wearing masks, washing hands, using sanitizers, and practicing social distancing.
2. Medical and health care aspects: this theme encompasses topics related to the medical and health care aspects of COVID-19, including hospitals, doctors, treatments, testing, and recovery.
3. Government and social measures: this theme covers government actions, social measures, and policies implemented to address the COVID-19 pandemic, including lockdowns, travel restrictions, home orders, suspending schools, avoiding gatherings, closing shops, staying at home, and support measures.
4. Impact and numbers: this theme involves discussions about the impact of COVID-19, including the number of cases, deaths, recoveries, and updates on the situation.
5. Vaccine development and research: this theme revolves around vaccine development, clinical trials, and scientific research related to finding a solution to COVID-19.
6. COVID-19 and religious practices: this topic discusses how COVID-19 has impacted religious practices and gatherings. It mentions places of worship (مسجد) and the importance of adhering to prayers (صلا) and religious guidelines (ستر) during the COVID-19 pandemic, especially during occasions such as Ramadan (رمضان). The theme also includes expressions of gratitude and good wishes for nations and people (سلام, خير, جمع).
7. Global impact of COVID-19 on sports and countries: this topic discusses the spread of COVID-19 in different countries, including China, Italy, and the United States, and its impact on various aspects, such as sports events and leagues in Europe and the Middle East. It also mentions the virus as a global pandemic and its effects on athletes and players (لاعب) as well as its presence in different regions around the world.
8. COVID-19 and national efforts: this theme focuses on the efforts of different nations, including Saudi Arabia and Kuwait, in combating COVID-19. It mentions leaders (حمد, سلمان, ملك) and their efforts to protect the health and well-being of their citizens (شعب, مواطن). The theme includes expressions of gratitude for the nation’s efforts in managing the COVID-19 pandemic (شكر) and highlights the importance of public health (صحه). Textbox 1 provides a comprehensive list of topics, thoughtfully translated into English for better clarity and accessibility.

**Table 4:** Themes based on topic classification, bigrams, and sample tweets.

Theme and topic			Bigrams		Sample tweets
**Preventive measures and safety**					
	Face mask	Wear mask		A note for your safety from the new coronavirus infection: Avoid social gatherings with more than 1 person. Avoid crowded areas or places where you might interact with individuals who are sick. Avoid handshakes as they are among the primary causes of virus transmission. Wear a mask whenever possible.	
	Hands	Wash hands, use sanitizers		Avoid gatherings, closed spaces, and crowded areas, along with regularly washing your hands with water and soap or sanitizing them with alcohol-based disinfectants. By God’s will, you will be protected from contracting the new coronavirus.	
	Social distancing	Social distancing		Social distancing means staying away from gatherings and crowded places. If you must leave your home, maintain a distance of at least 2 meters from the people around you. Source: Cleveland Clinic, COVID-19.	
**Medical and health care aspects**					
	Health authorities	Precautionary measures, followed the instructions		Home quarantine protects against the risk of a person spreading the coronavirus without showing symptoms, making them a potential source of transmission to various groups. Preventive measures against COVID-19 ease the burden on health care providers, enabling them to fulfill their roles in treating other illnesses and performing preventive tasks, including COVID-19 detection. Voice of the physician.	
	Recovery	Case recovery		Breaking: The Ministry of Health announces the recovery of the first coronavirus case in the kingdom. This concerns the young man who returned from Italy and was previously announced as the first imported case of the virus in Morocco. COVID-19, Morocco, Recovery, Ministry of Health.	
	Treatment	Treating the infected		The Minister of Health announces the initiation of treating patients with COVID-19 with the chloroquine vaccine.	
	Treatment	New drug		The *Washington Post* reports that Chinese experts and physicians have successfully fought COVID-19 using chloroquine, a drug primarily used to treat malaria, and Kaletra, an HIV medication that combines lopinavir and ritonavir. Emirati physician Omar Al Hammadi shares the success of this trial.	
	Hospital	Field hospital		Starting Sunday, a physician will accompany every ambulance, and a field hospital will be established inside the trade unions complex. Dr Ali Al-Abous, President of the Jordanian Medical Association, comments on the nationwide curfew in Jordan due to the COVID-19 pandemic.	
**Government and social measures**					
	Lockdowns and suspending	Closing shops, suspending schools		Precautionary measures in Kuwait against COVID-19: suspension of studies and work, cancellation of weddings, closure of mosques, closure of malls, closure of salons, partial curfew, extension of the suspension of studies, regulation of work in central markets, closure of shops, postponement of installments.	
	Travel restrictions	Travel ban		Saudi Arabia: Saudi Arabia suspended studies, banned cafes and shisha, prohibited sports gatherings and cinemas, halted entertainment activities, stopped Umrah and travel, and conducted intensive testing to search for patients. All for your benefit—help your government overcome these circumstances with minimal losses.	
	Home orders	Stay home		Stay home and protect your family from coronavirus. Prevention guidelines. Stay home.	
	Curfew	Curfew		Breaking: Al Jazeera correspondent reports the sounding of alarm sirens across Jordan as the nationwide curfew begins to combat the spread of COVID-19.	
	Remote	Remote work		It is everyone’s duty to follow the precautionary measures taken by our government, may God protect them, to prevent the spread of COVID-19. At our facility, we have informed the success team to work remotely from their homes until further notice.	
**Impact and numbers**					
	New cases	Confirmed cases, increase in cases		The Kuwaiti Ministry of Health has reported new cases of the novel coronavirus, and the total number of patients that have exited quarantine is 20.	
	Deaths	Coronavirus deaths		A new death has been recorded in Jordan due to COVID-19, bringing the total number of deaths to 5.	
**Vaccine development and research**					
	Religious guidelines	Prayer, supplication		Breaking: The Senior Scholars Authority calls on everyone to adhere to the instructions, guidelines, and regulations, to fear God, and to resort to prayer and supplication. COVID-19, Saudi Arabia.	
	Umrah	Suspension of Umrah		It was discovered during the COVID-19 crisis that preserving life is one of the most important objectives of Sharia, and everything is subordinated to it. The suspension of Umrah and prayer in mosques reflects the greatness of Islam and the depth of Sharia's objectives.	
**Global impact of COVID-19 on sports and countries**					
	Postponement of matches	Postponement of matches		The Union of European Football Association has decided to postpone all matches scheduled for next week. Sports, COVID-19.	
	Italy	The situation in Italy		Terrifying numbers in Italy and Iran; a video shows the spread of the coronavirus outside China until March.	
**COVID-19 and national efforts**					
	King Salman	Royal support		King Salman bin Abdulaziz and Crown Prince Mohammed bin Salman. The Saudi Arabian Monetary Authority announces support for the private sector with 1 billion Saudi riyals to face the expected financial and economic impacts of the coronavirus.	
	Thanks	Government gratitude		We thank God for the blessing of Islam and the blessing of Salman. Every Saudi has the right to be proud and boast about Saudi Arabia. May God protect its government and people from all harm. Saudi Arabia. COVID-19. Stay at home.

**Topic and words (English translations) used in the study.**

- Topic 0: country, corona, Kuwait, Hamad, preserve, Alhamdulillah, blessing, people, preserve, people, state, good, world, Saudi Arabia, Muslim, disease, thanks, epidemic, virus, and Egypt
- Topic 1: corona, calamity, epidemic, good, virus, Muslim, mercy, prayer, new, by, world, lift, great, disease, heart, raise, evil, people, mercy, and land
- Topic 2: corona, hand, virus, mask, wash, people, new, water, sanitizer, road, discount, know, world, wear, person, soap, use, gloves, mask, and beautiful
- Topic 3: corona, virus, disease, Iran, medical, infected, hospital, doctor, treatment, Iraq, test, health, person, device, hospital, Bahrain, infected, transfer, Italy, and system
- Topic 4: corona, virus, Kuwait, Egypt, new, novel, Covid, health, visit, suspension, Saudi Arabia, core, statement, Kuwaiti, confront, Emirate, study, cure, country, and protection
- Topic 5: corona, virus, condition, new, condition, infected, health, infected, and, infection, urgent, registration, death, announce, visit, rise, recovery, register, total, and rise
- Topic 6: corona, virus, education, visit, minister, confront, support, special, health, student, bank, publish, sector, state, official, crisis, contact, community, request, house
- Topic 7: corona, China, country, virus, world, epidemic, union, Italy, football, Europe, league, America, new, spread, foot, player, and east
- Topic 8: corona, virus, home, scene, protect, country, Algeria, Egypt, die, wind, detail, video, top, people, young, Morocco, new, picture, wanted, and film
- Topic 9: home, corona, stay, ban, quarantine, circulation, homely, virus, new, Saudi Arabia, home, Kuwait, official, activity, circulation, health, wanted, complete, good, and old
- Topic 10: corona, virus, world, Trump, Oman, new, vaccine, president, confront, America, China, treatment, wanted, news, Chinese, company, vaccine, coveted, American, and Palestine
- Topic 11: corona, virus, spread, health, state, epidemic, protection, disease, enemy, awareness, threat, road, gathering, mandatory, country, avoid, citizen, world, and protection
- Topic 12: corona, mosque, people, gathering, prayer, congregation, Lebanon, condition, Ramadan, virus, prayer, I mean, talk, cover, world, Egypt, great, good, people, and peace
- Topic 13: corona, virus, procedure, spread, prevention, decision, sanitization, closure, local, logic, visit, urgent, Saudi Arabia, new, governor, application, shop, Riyadh, precautionary, and system
- Topic 14: corona, Saudi Arabia, thanks, Kuwait, king, health, preserve, country, homeland, virus, citizen, people, visit, effort, state, crisis, blessing, Salman, epidemic, and preserve
- Topic 15: corona, Lebanon, people, and, age, came out, quarantine, from me, subject, condition, house, and, mean, virus, peace, health, Egypt, people, world, and damn

### Sentiment Analysis

**Figure 4:**
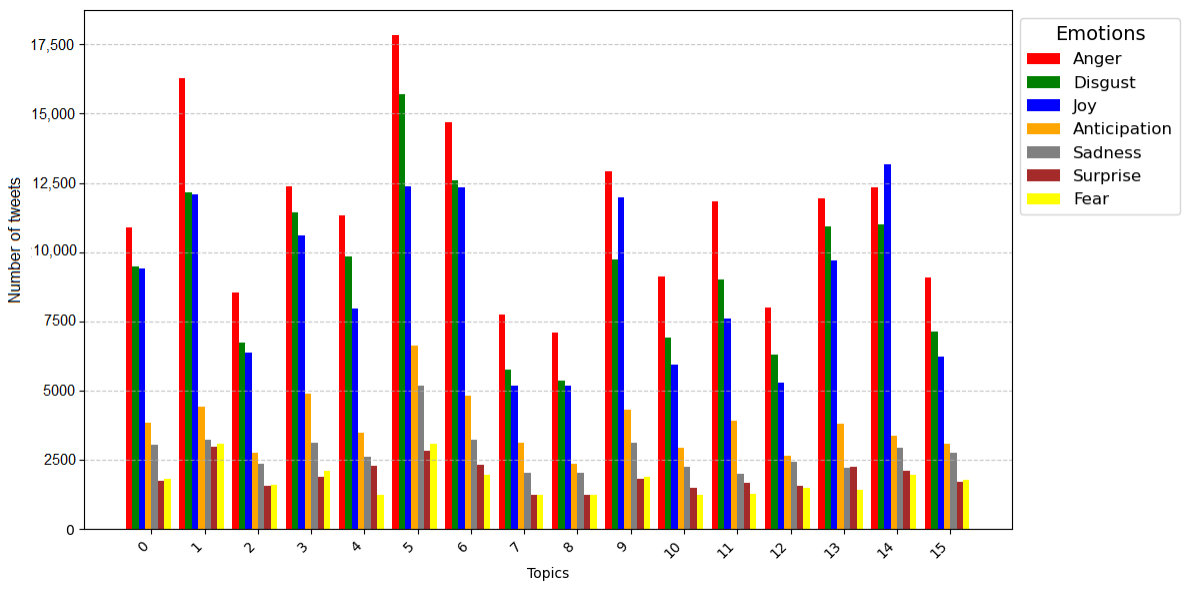
Sentiment analysis for each of the 16 latent topics.

We conducted sentiment analysis for each of the 16 topics and presented the results in Figure 4 and Table 5. Figure 4 visualized 7 emotions: anger, disgust, joy, anticipation, sadness, surprise, and fear. Across all 16 topics, anger (represented by the red line) was the dominant emotion in 16 topics, followed by disgust (green line), joy (blue line), and anticipation (orange line). To delve deeper into the emotional aspects of the data, we provide a breakdown of the number of tweets associated with each emotion across different topics in Table 5. For example, in topic 5, a substantial number of tweets (n=17,848) expressed anger, reflecting a strong sentiment regarding the need for essential measures and precautions. This high prevalence of anger in topic 5 stands out in comparison to the other topics. It is worth noting that excessive anger, if left unmanaged, can lead to a range of medical problems. Managing emotions such as anger is crucial not only for mental well-being but also for overall physical health.

**Table 5:** The number of tweets for 7 emotions across 16 topics.

Topic	Anger	Anticipation	Disgust	Fear	Joy	Sadness	Surprise
0	10,896	3834	9475	1835	9428	3040	1757
1	16,295	4434	12,164	3079	12,083	3217	2965
2	8559	2772	6729	1585	6366	2346	1554
3	12,391	4894	11,424	2110	10,602	3113	1876
4	11,345	3493	9838	1246	7969	2624	2295
5	17,848	6630	15,688	3078	12,375	5192	2847
6	14,702	4820	12,601	1979	12,329	3239	2342
7	7757	3130	5752	1224	5189	2031	1230
8	7082	2367	5344	1222	5168	2035	1228
9	12,926	4321	9742	1896	11,989	3132	1802
10	9113	2948	6901	1225	5953	2245	1486
11	11,836	3915	9027	1288	7606	2015	1656
12	7988	2646	6286	1494	5282	2416	1547
13	11,942	3801	10,928	1437	9717	2231	2241
14	12,328	3357	11,007	1962	13,172	2953	2124
15	9097	3087	7116	1779	6218	2762	1716

## Discussion

### Principal Findings

This study delved into public discussion and emotional expressions related to COVID-19 using Arabic Twitter messages. Twitter users engaged in discussions encompassing 8 primary themes regarding COVID-19. Using topic modeling on the tweets proved valuable in uncovering insights into COVID-19–related topics and concerns. The outcomes highlighted several crucial observations.

This analysis concentrates on tweets from March 2020, a pivotal phase in the COVID-19 pandemic’s unfolding narrative. During this period, the second stage of the COVID-19 pandemic emerged prominently, marked by a significant milestone as Arabic countries reported their initial cases of COVID-19. Subsequently, a cascade of vital health measures ensued, encompassing the enforcement of quarantine protocols, the temporary cessation of air travel, and the inevitable postponement or cancelation of various events. This time frame aligns logically with the peak frequency of tweets, as previously observed by Taneja et al [22] and Haouari et al [34].

Amidst the array of all 16 topics, a discernible pattern surfaced, characterized by the recurring presence of specific keywords such as “coronavirus,” “increase,” “health,” “new,” “infected,” “death,” “recovery,” and “case.” This linguistic cluster strongly implies a surge in COVID-19 infections, accompanied by lamentable loss of life and the emergence of new cases during the ongoing COVID-19 pandemic. It is imperative to emphasize that our chosen time frame aligns precisely with the onset of the COVID-19 pandemic’s second phase, coinciding with heightened global concern. The substantial spike in COVID-19 cases in Italy during this period ignited a profound sense of alarm on a global scale. This surge in worldwide apprehension may have contributed to the observed increase in tweet frequency, corroborating findings from multiple studies [22,34].

Furthermore, substantial discussions revolving around the COVID-19 pandemic within diverse Arabic nations have drawn significant interest. These conversations are marked by a prevailing sense of indignation. Moreover, public sentiments concerning the spread of COVID-19 unveiled an underlying sense of anticipation toward prospective measures. These sentiments were accompanied by a mix of emotions, including anger and fear; a notable undercurrent of fear was predominant in discussions revolving around the COVID-19 crisis and the resulting fatalities. This trend aligns with global sentiments, as documented by Lwin et al [36], wherein public emotions underwent a noticeable shift from fear to anger throughout the COVID-19 pandemic, with traces of sadness and joy also emerging.

Noteworthy, the appearance of dialogues concerning COVID-19 and religious practices introduced a fresh subject not previously detected in prior research. This indicates a developing connection between COVID-19 and religious matters on the Twitter platform. This is particularly apparent due to the substantial influence of religious identity on attitudes and actions concerning the COVID-19 pandemic and vaccination efforts; the COVID-19 pandemic has significantly reshaped communal worship and gatherings as measures to curb the virus’s transmission [37]. Furthermore, religious leaders have assumed a central role in championing COVID-19 vaccination campaigns, effectively addressing and mitigating vaccine hesitancy [38].

### In-Depth Analysis of Findings

The application of topic modeling and sentiment analysis in this study provided several valuable insights into public sentiment and thematic discussions during the early stages of the COVID-19 pandemic in Arab regions. The findings largely align with anticipated outcomes, such as the focus on preventive measures and safety and medical and health care aspects, both of which were expected topics given the nature of the COVID-19 pandemic.

However, the emergence of discussions on COVID-19 and religious practices was a unique finding that adds depth to the understanding of public discourse in Arab communities. This theme highlights the intersection of the COVID-19 pandemic with cultural and religious practices, which had not been as thoroughly explored in previous research. It underscores the significant impact that COVID-19 had on religious identity, communal worship, and adherence to religious guidelines during pivotal periods such as Ramadan.

Another notable aspect was the attention given to the global impact of COVID-19 on sports and countries, reflecting the broad international concern and how global events, especially sports, were affected. This indicates that the COVID-19 pandemic’s influence went beyond public health and extended into societal and cultural dimensions, impacting activities that are deeply integrated into daily life.

In addition, the sentiment analysis revealed a nuanced distribution of emotions, with a significant proportion of tweets expressing anger and disgust, as expected, given the uncertainty surrounding the COVID-19 pandemic. However, there was also a notable presence of positive emotions, such as hope and solidarity, particularly in tweets discussing community support and coping mechanisms. This suggests that, despite the overwhelming nature of the crisis, many users turned to social media not only to express negative emotions but also to share supportive messages and encourage others.

Overall, the identified themes and their respective discussions provide a comprehensive view of public sentiment, concerns, and priorities during the early COVID-19 pandemic period. These insights not only reflect the immediate response to the health crisis but also highlight the diverse and context-specific aspects that shaped public discourse. Such findings offer a foundation for more effective public health communication and intervention strategies, particularly in culturally sensitive contexts.

### Strengths

This study provided valuable insights into the sentiments and concerns of Arabic-speaking Twitter users during the COVID-19 pandemic, underscoring the significance of social media as a means of understanding and addressing public health issues in the digital era. First, the analysis encompassed a substantial dataset of 1 million Arabic tweets, offering a comprehensive view of the sentiments and topics expressed by Twitter users in Arabic-speaking countries during a specific period of the COVID-19 pandemic. Besides, the study used a combination of machine learning techniques, including topic modeling and sentiment analysis, to uncover and categorize themes and emotions within the dataset, providing a holistic understanding of the data. By identifying and categorizing 16 conversation topics into 8 themes, the study offered a structured view of the discussions surrounding COVID-19 in the Arab region, making it easier to interpret and use the findings. Finally, the inclusion of emotion analysis adds depth to the study, revealing how Twitter users in the Arab world emotionally responded to various aspects of the COVID-19 pandemic.

### Limitations

First, at the forefront of our approach, we meticulously aimed to unravel the complexities embedded within the COVID-19 pandemic’s second phase. Our focus was sharp and exclusive, centered on harnessing tweets originating exclusively from March 2020. The motivation behind this specific time frame stemmed from our intention to subject translated tweets to a comprehensive sentiment analysis. This intricate process relied upon the Google API translation service, which, although effective, is accompanied by a substantial cost factor. The financial implication associated with translating the entirety of the datasets using this service was a noteworthy consideration that prompted us to make strategic choices in our analysis approach.

Second, it is crucial to recognize that Arabic is a linguistically intricate language characterized by a rich array of dialects and intricate cultural nuances. These unique linguistic qualities can present substantial challenges for automated sentiment analysis tools. While we attempted to apply automated sentiment analysis to Arabic tweets, we encountered difficulties in precisely capturing the subtleties of emotions. Automated tools often grappled with interpreting nuanced sentiments, such as sarcasm, irony, and contextual shifts in sentiment that frequently permeate social media conversations.

Third, a strategic decision was made to exclude non-Arabic tweets from our analyses. As a result, our findings were inherently confined to users who exclusively communicated in Arabic. It is essential to underscore that the fundamental objective of our research revolves around gaining insights into the opinions and reactions of Arabic countries in relation to COVID-19.

Furthermore, while our study leveraged social media data as a proxy for public sentiment, it is essential to recognize the inherent biases associated with using Twitter data. For instance, social media users may not be representative of the general population, as certain demographics might be underrepresented on platforms such as Twitter. A study by Padilla et al [39] has shown that social media content can be biased based on whether individuals are local residents or visitors and the types of activities they engage in throughout the day. Similarly, Gore et al [40] highlighted that the sentiment of tweets is often correlated with the geographical area in which they were composed, suggesting that local context and specific events may have a significant impact on sentiment analysis results. Frank et al [41] also found that emotional expressions, such as happiness, vary significantly by location, further reinforcing the influence of geographic factors on sentiment.

In addition, it is plausible that individual personality traits or political affiliations, as suggested by Auer and Elena [42], could influence whether a user expresses positive or negative sentiments. This raises an open question about the extent to which sentiment reflects variance in psychological traits versus the situational context in which those traits are expressed. These factors could contribute to biases in our dataset and should be considered as potential sources of influence on the study’s outcomes.

### Future Work

Regarding future studies focusing on COVID-19, first, there arises a noteworthy avenue for exploration comparing the sentiments and opinions of Arabic-speaking populations with those of individuals expressing themselves in other languages. A comprehensive approach might encompass languages such as English, Italian, French, German, and Spanish. Such comparative analyses have the potential to yield valuable insights into the cross-linguistic dynamics of perceptions and responses to the COVID-19 pandemic.

Second, another promising avenue for future research involves conducting a comparative analysis between sentiment analysis using human-labeled data and automated tools specifically tailored for Arabic languages. This comparative study should aim to ascertain the feasibility of leveraging these automated tools as an alternative to translation APIs. By meticulously comparing the results obtained from human-labeled sentiment analysis and those generated by automated tools, researchers can gauge the efficacy, accuracy, and reliability of automated sentiment analysis for Arabic tweets. The outcomes of this research hold the potential for far-reaching implications, potentially presenting a cost-effective and streamlined avenue for sentiment analysis that eliminates the reliance on costly translation APIs.

By providing an accurate and efficient mechanism for measuring sentiments in Arabic tweets, researchers and mental health professionals could identify patterns of emotional distress or psychological well-being. This could be especially pertinent during times of crises, enabling timely interventions and support for individuals experiencing heightened emotional responses. Importantly, the ability to effectively harness sentiment analysis for understanding emotional states has the potential to empower the broader field of mental health research and intervention as well as enhance our understanding of collective emotional dynamics within Arabic-speaking communities.

Third, there is an imminent need for research to unravel the stem of fabricated tweets that emerge during a pandemic. Given that Twitter users experience a heightened sense of fear, which might be exacerbated by the proliferation of misinformation, it becomes a critical endeavor to investigate the prevalence and impact of false tweets. Subsequent studies could significantly benefit from spotlighting the issue of misinformation, with a specific focus on understanding how government officials and international organizations can effectively manage the dissemination of deceptive messages targeting the public. By comprehensively addressing the challenges posed by misleading content, we can enhance our collective understanding of navigating information dissemination during such critical periods.

### Conclusions

This study delves deep into the intricate web of topics and emotions found in Arabic tweets about COVID-19. It highlights how platforms such as Twitter, especially during times of global change, are crucial for capturing the diverse feelings and concerns of Arabic speakers. Through a mix of topic modeling and sentiment analysis, we revealed the basic human emotions in user responses to COVID-19 tweets from March 2020.

We used 2 methods together: topic modeling (specifically LDA) and sentiment analysis tools. These helped us uncover the main themes and feelings within the tweets. Anger was the prominent emotion tied to COVID-19 topics, accompanied by other emotions. Joy was linked to vaccine and education discussions, while authority and politics stirred up anger. Sadness emerged from topics about cases, deaths, and the impacts on families and mental health.

This study connects social media, emotions, and the global scene. It sheds light on the emotional layers of digital conversations, offering insights into COVID-19–related tweets. These findings guide better communication strategies and compassionate responses, strengthening our collective resilience in the face of challenges.

Moreover, the results and workflow of this study present actionable insights for the medical and public health communities. By integrating our findings into official government documentation or public health research, authorities can tailor their communication strategies based on public concerns and emotions. This, in turn, helps in shaping more effective educational campaigns and policy interventions. Our methodology also serves as a robust tool for continuous monitoring of public sentiment in real time, allowing policy makers to stay informed and adapt their strategies accordingly. This approach ensures that responses are not only timely but also grounded in the actual sentiments and needs of the population.

## Appendix

Multimedia Appendix 1 Original Arabic versions of tweets shown in Table 2.

Multimedia Appendix 2 Original Arabic versions of tweets shown in Table 3.

Multimedia Appendix 3 Original Arabic versions of tweets shown in Table 4.

## Abbreviations

API: application programming interface
LDA: latent Dirichlet allocation
NLP: natural language processing

## Data Availability

The data sets generated and analyzed during this study are available in the GitHub repository [43].
