# Supplementary material for: Unveiling Topics and Emotions in Arabic Tweets Surrounding the COVID-19 Pandemic: Topic Modeling and Sentiment Analysis Approach
Source: JMIR Infodemiology. 2025 Feb 10;5:e53434. doi: 10.2196/53434 (PMC11851025; doi:10.2196/53434)
Supplement: Multimedia Appendix 1 [file infodemiology_v5i1e53434_app1.docx]

**Multimedia Appendix 1**

**Table 2.** Top 50 unigrams and bigrams and their distributions.

|  | | Values (%) |
| --- | --- | --- |
| **Top 50 unigrams** | | |
|  | كورونا (Coronavirus) | 6.558451 |
|  | فيروس (Virus) | 2.350919 |
|  | بيت (Home) | 0.921041 |
|  | جديد (New) | 0.857981 |
|  | صحه (Health) | 0.614924 |
|  | كويت (Kuwait) | 0.576566 |
|  | حال (Condition) | 0.551307 |
|  | السعوديه (Saudi Arabia) | 0.503562 |
|  | عالم (World) | 0.491143 |
|  | دولة (Country) | 0.487031 |
|  | زار (Visit) | 0.392251 |
|  | وباء (Pandemic) | 0.391468 |
|  | حظر (Curfew) | 0.359459 |
|  | خليك (Stay) | 0.359077 |
|  | بلد (Country) | 0.352204 |
|  | انتشار (Spread) | 0.34872 |
|  | أصاب (Infected) | 0.340486 |
|  | حجر (Quarantine) | 0.339662 |
|  | حالة (Case) | 0.335292 |
|  | مرض (Disease) | 0.331376 |
|  | مصاب (Infected) | 0.328934 |
|  | عاجل (Urgent) | 0.314949 |
|  | مصر (Egypt) | 0.313753 |
|  | فايروس (Virus) | 0.288958 |
|  | ناس (People) | 0.272675 |
|  | وزير (Minister) | 0.263771 |
|  | شعب (People) | 0.257506 |
|  | صحي (Health) | 0.244108 |
|  | صين (China) | 0.243201 |
|  | خير (Good) | 0.241965 |
|  | تجول (Travel) | 0.241181 |
|  | مواطن (Citizen) | 0.239945 |
|  | كوفيد (COVID) | 0.238966 |
|  | ملك (King) | 0.238255 |
|  | مستجد (New) | 0.220993 |
|  | إجراء (Procedure) | 0.213274 |
|  | لبنان (Lebanon) | 0.211883 |
|  | أراد (Wanted) | 0.209183 |
|  | مواجه (Confrontation) | 0.205782 |
|  | تعليم (Education) | 0.205174 |
|  | وفى (In) | 0.198331 |
|  | إصابة (Infection) | 0.193302 |
|  | شكر (Thanks) | 0.187623 |
|  | أعلن (Announced) | 0.186263 |
|  | منع (Prevention) | 0.185222 |
|  | وطن (Nation) | 0.184861 |
|  | إيران (Iran) | 0.180255 |
|  | منزل (House) | 0.178111 |
|  | ايطاليا (Italy) | 0.174504 |
|  | منزلي (In house) | 0.172979 |
| **Top 50 bigrams** | | |
|  | فيروس, كورونا (Virus, coronavirus) | 2.029932 |
|  | كورونا, جديد (Coronavirus, new) | 0.526419 |
|  | خليك, بيت (Stay home) | 0.325347 |
|  | كورونا, كورونا (Coronavirus, coronavirus) | 0.302665 |
|  | زار, صحه (Visit, health) | 0.263658 |
|  | فايروس, كورونا (Virus, coronavirus) | 0.19593 |
|  | كورونا, كويت (Coronavirus, Kuwait) | 0.194992 |
|  | كورونا, مستجد (Coronavirus, new) | 0.192446 |
|  | حظر, تجول (Curfew, travel) | 0.18009 |
|  | انتشار, فيروس (Spread, virus) | 0.155542 |
|  | كورونا, فيروس (Coronavirus, virus) | 0.146133 |
|  | حجر, منزلي (Quarantine, home) | 0.138868 |
|  | حجر, صحي (Quarantine, health) | 0.123512 |
|  | جديد, فيروس (New, virus) | 0.122492 |
|  | كورونا, لبنان (Coronavirus, Lebanon) | 0.108992 |
|  | وباء, كورونا (Pandemic, coronavirus) | 0.108868 |
|  | بيت, كورونا (Home, coronavirus) | 0.107683 |
|  | كورونا, السعوديه (Coronavirus, Saudi Arabia) | 0.105704 |
|  | كورونا, مصر (Coronavirus, Egypt) | 0.103818 |
|  | مصاب, فيروس (Infected, virus) | 0.102376 |
|  | أصاب, جديد (New, case) | 0.09342 |
|  | كورونا, كوفيد (Coronavirus, COVID) | 0.091503 |
|  | كويت, كورونا (Kuwait, coronavirus) | 0.089236 |
|  | جديد, كورونا (New, coronavirus) | 0.088587 |
|  | صحه, العالميه (Health, global) | 0.08464 |
|  | خلك, بيت (Stay, home) | 0.083898 |
|  | وزير, صحه (Minister, health) | 0.083743 |
|  | ازم, كورونا (Crisis, coronavirus) | 0.083589 |
|  | كورونا, خليك (Coronavirus, stay) | 0.076416 |
|  | منظم, صحه (Organizer, health) | 0.073128 |
|  | مواجه, كورونا (Confrontation, coronavirus) | 0.068563 |
|  | حال, وفى (Condition, in) | 0.06845 |
|  | السعوديه, كورونا (Saudi Arabia, coronavirus) | 0.064812 |
|  | كورونا, أراد (Coronavirus, wanted) | 0.061967 |
|  | كورونا, عاجل (Coronavirus, urgent) | 0.060535 |
|  | تسجيل, حال (Recording, case) | 0.055537 |
|  | مواجه, فيروس (Confrontation, virus) | 0.054918 |
|  | تفشي, فيروس (Spread, virus) | 0.053424 |
|  | انتشار, كورونا (Spread, coronavirus) | 0.053187 |
|  | كورونا, حظر (Coronavirus, curfew) | 0.050755 |
|  | حظر, تجوال (Curfew, curfew) | 0.04958 |
|  | إجراء, احترازيه (Procedure, precautionary) | 0.049426 |
|  | ولاية, المتحده (United, State) | 0.048818 |
|  | بقاء, منزل (Staying, home) | 0.048519 |
|  | مرض, كورونا (Disease, coronavirus) | 0.047993 |
|  | مصاب, كورونا (Infected, coronavirus) | 0.047849 |
|  | مواطن, مقيم (Citizen, resident) | 0.047684 |
|  | خادم, حرم (Servant, holy mosque) | 0.04552 |
|  | منع, تجول (Prevention, travel) | 0.045458 |
|  | كورونا, زار (Coronavirus, visit) | 0.044582 |
