# Supplementary material for: Unveiling Topics and Emotions in Arabic Tweets Surrounding the COVID-19 Pandemic: Topic Modeling and Sentiment Analysis Approach
Source: JMIR Infodemiology. 2025 Feb 10;5:e53434. doi: 10.2196/53434 (PMC11851025; doi:10.2196/53434)
Supplement: Multimedia Appendix 2 [file infodemiology_v5i1e53434_app2.docx]

**Multimedia Appendix 2**

**Table 3.** Topic, words, and percentage of tweets.

| Topic | Words | Values (%) |
| --- | --- | --- |
| 0 | بلد (country), كورونا (corona), كويت (Kuwait), حمد (praise), حفظ (protection), حمدله (gratitude to god), نعمة (blessing), أهل (people), احفظ (protect), شعب (people or nation), دولة (state), خير (goodness), عالم (world), السعوديه (Saudi Arabia), مسلم (Muslim), مرض (illness), شكر (thanks), وباء (pandemic), فايروس (virus), مصر (Egypt) | 6.31 |
| 1 | كورونا (corona), بلاء (affliction), وباء (pandemic), خير (goodness), فايروس (virus), مسلم (Muslim), رحم (mercy), دعاء (supplication/prayer), جديد (new), بال (mind), عالم (world), رفع (lift or remove), عظيم (great), مرض (illness), قلب (heart), أرفع (raise), شر (evil), ناس (people), رحمة (mercy), أرض (Earth) | 8.5 |
| 2 | كورونا (corona), يد (hand), فايروس (virus), كمام (mask), غسل (washing), ناس (people), جديد (new), ماء (water), معقم (sanitizer), طريق (way), خصم (discount), عرف (knowledge), عالم (world), لبس (wear), شخص (person), صابون (soap), استخدام (usage), قفاز (glove), كمامه (mask), جميل (beautiful) | 4.69 |
| 3 | كورونا (corona), فايروس (virus), مرض (illness), إيران (Iran), طبي (medical), مصاب (infected), مستشفى (hospital), طبيب (doctor), علاج (treatment), عراق (Iraq), فحص (examination or test), صحي (health), شخص (person), جهاز (device), مستشفي (hospital), بحرين (Bahrain), اصاب (infected), نقل (transmission), ايطاليا (Italy), نظام (system) | 7.28 |
| 4 | كورونا (corona), فايروس (virus), كويت (Kuwait), مصر (Egypt), جديد (new), مستجد (emerging), كوفيد (COVID), صحه (health), زار (visited), تعليق (suspension), السعوديه (Saudi Arabia), كورو (corona), بيان (statement), كويتي (Kuwaiti), تصدي (confrontation), إمارة (Emirate), دراسه (study), كرو (crew), دولة (state), وقاي (prevention) | 6.09 |
| 5 | كورونا (corona), فايروس (virus), حال (condition), جديد (new), حالة (case), أصاب (infected), صحه (health), مصاب (infected), وفى (died), إصابة (infection), عاجل (urgent), تسجيل (recording), وفاة (death), أعلن (announced), زار (visited), ارتفاع (increase), شفاء (recovery), سجل (recorded), إجمالي (total), ارتفع (rose) | 9.98 |
| 6 | كورونا (corona), فايروس (virus), تعليم (education), زار (visited), وزير (minister), مواجه (confrontation), دعم (support), خاص (private), صحه (health), طالب (student), بنك (bank), نشر (spread), قطاع (sector), دولة (state), مسءول (responsible), ازم (crisis), تواصل (communication), مجتمع (community), طلب (request), منزل (home) | 8.16 |
| 7 | كورونا (corona), صين (China), دولة (state), فايروس (virus), عالم (world), وباء (pandemic), اتحاد (union), ايطاليا (Italy), كره (hate), أوروبا (Europe), دوري (league), أمريكا (America), جديد (new), انتشار (spread), قدم (presented), حال (condition), أوربي (European), فعل (action), لاعب (player), شرق (east) | 4.13 |
| 8 | كورونا (corona), فايروس (virus), دار (house), مشهد (scene), احمي (protect), بلد (country), جزائر (Algeria), مصر (Egypt), مات (died), ريح (rest), تفصيل (detail), فيديو (video), نتصدر (lead), ناس (people), شاب (young man), مغرب (Morocco), جديد (new), صورة (image), أراد (wanted), فيلم (film) | 3.83 |
| 9 | بيت (house), كورونا (corona), خليك (stay), حظر (curfew), حجر (quarantine), تجول (wandering), منزلي (home based), فايروس (virus), جديد (new), السعوديه (Saudi Arabia), منزل (home), كويت (Kuwait), مسءول (responsible), فعالية (effectiveness), تجوال (roaming), صحي (health), أراد (wanted), كامل (complete), خير (goodness), زم (Zoom) | 7.18 |
| 10 | كورونا (corona), فايروس (virus), عالم (world), ترامب (Trump), عمان (Oman), جديد (new), لقاح (vaccine), رئيس (president), واجه (faced), أمريكا (America), صين (China), علاج (treatment), أراد (wanted), اخبار (news), صيني (Chinese), شرك (partnership), قاح (vaccine), كوفيد (COVID), أمريكي (American), فلسطين (Palestine) | 4.68 |
| 11 | كورونا (corona), فايروس (virus), انتشار (spread), صحه (health), دولة (state), وباء (pandemic), وقايه (prevention), مرض (illness), عدو (enemy), وعي (awareness), خطر (danger), طريق (way), تجمع (gathering), وجب (must), منزل (home), بلد (country), تجنب (avoidance), مواطن (citizen), عالم (world), وقاي (prevention) | 5.86 |
| 12 | كورونا (corona), فايروس (virus), انتشار (spread), صحه (health), دولة (state), وباء (pandemic), وقايه (prevention), مرض (illness), عدو (enemy), وعي (awareness), خطر (danger), طريق (way), تجمع (gathering), وجب (must), منزل (home), بلد (country), تجنب (avoidance), مواطن (citizen), عالم (world), وقاي (prevention) | 5.86 |
| 13 | كورونا (corona), صين (China), دولة (state), فايروس (virus), عالم (world), وباء (pandemic), اتحاد (union), ايطاليا (Italy), كره (hate), أوروبا (Europe), دوري (league), أمريكا (America), جديد (new), انتشار (spread), قدم (presented), حال (condition), أوربي (European), فعل (action), لاعب (player), شرق (east) | 4.13 |
| 14 | كورونا (corona), السعوديه (Saudi Arabia), شكر (thanks), كويت (Kuwait), ملك (king), صحه (health), حفظ (protection), بلد (country), وطن (homeland), فايروس (virus), مواطن (citizen), شعب (people or nation), زار (visited), جهد (effort), دولة (state), حرم (sanctuary), حمد (praise), سلمان (Salman), وباء (pandemic), احفظ (protect) | 7.35 |
| 15 | كورونا (corona), لبنان (Lebanon), ناس (people), ون (one), عمر (age), طلع (went out), حجر (quarantine), عنى (meant), موضوع (topic), حال (condition), بيت (house), يش (what), فايروس (virus), سلم (safety), صحي (health), مصر (Egypt), شعب (people or nation), دنيا (world) | 4.98 |
