# Supplementary material for: Unveiling Topics and Emotions in Arabic Tweets Surrounding the COVID-19 Pandemic: Topic Modeling and Sentiment Analysis Approach
Source: JMIR Infodemiology. 2025 Feb 10;5:e53434. doi: 10.2196/53434 (PMC11851025; doi:10.2196/53434)
Supplement: Multimedia Appendix 3 [file infodemiology_v5i1e53434_app3.docx]

**Multimedia Appendix 3**

**Table 4.** Themes based on topic classification, bigrams, and sample tweets.

| Theme and topic | | Bigrams | Sample tweets |
| --- | --- | --- | --- |
| **Preventive measures and safety** | | | |
|  | الكمامة (Face mask) | البس الكمام (Wear mask) | لفته لسلامتك من عدوى فيروس كورونا الجديد تجنب المناسبات الاجتماعية التي يوجد بها أكثر من شخص تجنب المناطق المزدحمة أو الأماكن التي يمكن أن تتعامل فيها مع أشخاص مرضى تجنب المصافحة حيث أنها من أكثر أسباب انتقال العدوى البس الكمام ما استطعت. |
|  | اليدين (Hands) | غسل اليدين، استخدام المعقم (Wash hands, use sanitizers) | تجنبك التجمعات والأماكن المغلقة والمزدحمة والمواظبة على غسل اليدين بشكل مستمر بالماء والصابون أو تعقيمها باستخدام المعقمات الكحولية تقيك بإذن الله من الإصابة بفيروس كورونا الجديد. |
|  | التباعد الاجتماعي (Social distancing) | مسافات متباعدة (Social distancing) | التباعد الاجتماعي هو الابتعاد عن التجمعات والأماكن المزدحمة وإذا اضطررت للخروج من بيتك حافظ على مسافة مترين على الأقل ممن حولك من الناس. المصدر: كليفلاند كلينيك فيروس كورونا كورونا. |
| **Medical and health care aspects** | | | |
|  | الجهات الصحية (Health authorities) | الإجراءات الاحترازية، التزام التعليمات (Precautionary measures, followed the instructions) | الحجر المنزلي يقي من خطر إصابة شخص بفيروس كورونا دون ظهور الأعراض عليه، مما يجعله وسيلة لنقل الفيروس لتجمعات مختلفة. الوقاية من كورونا تخفف العبء على الجهات الصحية لتتمكن من القيام بدورها في علاج باقي الأمراض وأدوارها الوقائية ومنها الكشف عن كوفيد. صوت الطبيب. |
|  | شفاء (Recovery) | شفاء الحالة (Case recovery) | عاجل: وزارة الصحة تعلن شفاء الحالة الأولى المصابة بفيروس كورونا بالمملكة. يتعلق الأمر بالشاب القادم من إيطاليا والذي علن في وقت سابق كأول حالة وافدة للإصابة بالفيروس بالمغرب. كورونا فيروس المغرب شفاء. وزارة الصحة. |
|  | معالجة (Treatment) | معالجة المصابين (Treating the infected) | وزير الصحة يعلن الشروع في معالجة المصابين بفيروس كورونا بلقاح كلوروكين. |
|  | علاج (Treatment) | عقار جديد (New drug) | واشنطن بوست الخبراء الصينيون والأطباء نجحوا في محاربة كورونا بعقار الكلوروكين وهو عقار يستخدم بشكل رئيسي لمعالجة مرض الملاريا وعقار مضاد لفيروس نقص المناعة البشرية الإيدز كاليترا وهو مزيج من لوبينافير وريتونافير الدكتور الإماراتي عمر الحمادي ينقل لكم نجاح التجربة. |
|  | مستشفى (Hospital) | مستشفى ميداني (Field hospital) | اعتباراً من الأحد طبيب مع كل سيارة إسعاف ومستشفى ميداني داخل مجمع النقابات نقيب الأطباء علي العبوس للمملكة حظر التجول كورونا الأردن حكومة الظل هنا المملكة. |
| **Government and social measures** | | | |
|  | تعليق, الإغلاق (Lockdowns and suspending) | اغلاق الأسواق, تعليق الدراسة (Closing shops, suspending schools) | الخطوات الاحترازية في الكويت تجاه كورونا تعليق الدراسة والعمل إلغاء الأعراس اغلاق المساجد اغلاق المجمعات اغلاق الصالونات الحظر الجزئي تمديد تعليق الدراسة تنظيم عمل الأسواق المركزية اغلاق المحلات تأجيل الأقساط. |
|  | قيود السفر (Travel restrictions) | منع السفر (Travel ban) | السعودية كورونا السعودية علقت الدراسة منعت المقاهي والشيشة ومنعت التجمهر الرياضي وكذلك السينما ايضاً وقفت فعاليات ترفيهية وقفت العمرة والسفر أيضاً والكشف المكثف للبحث عن المرضى كله عشان مصلحتك ساعد حكومتك بتخطي هالظروف بأقل خسائر. |
|  | البقاء في البيت (Home orders) | في البيت (Stay home) | ألتزم البقاء في البيت وحافظ على عائلتك من فيروس كورونا. أرشادات الوقاية. اجلس بالبيت. |
|  | حظر (Curfew) | حظر تجوال (Curfew) | عاجل: مراسل الجزيرة إطلاق صفارات الإنذار في أنحاء الأردن مع بدء حظر تجوال عام لمواجهة تفشي فيروس كورونا. |
|  | عن بعد (Remote) | العمل عن بعد (Remote work) | واجب على الجميع أتباع الإجراءات الوقائية الاحترازية المتخذة من حكومتنا حفظهم الله لمنع انتشار فيروس كورونا ونحن في منشأتنا أبلغنا فريق النجاح بالعمل عن بعد من منازلهم حتى إشعار آخر. |
| **Impact and numbers** | | | |
|  | حالات جديدة (New cases) | ارتفاع المصابين، حالات مؤكدة (Confirmed cases, increase in cases) | وزارة الصحة الكويتية تسجيل إصابات جديدة بفيروس كورونا المستجد وإجمالي عدد الحالات التي خرجت من الحجر الصحي 20 حالة. |
|  | الوفيات (Deaths) | وفيات كورونا (Coronavirus deaths) | تسجيل حالة وفاة جديدة في الأردن بسبب فيروس كورونا ليرتفع العدد الإجمالي إلى 5 وفيات. |
| **Vaccine development and research** | | | |
|  | التوجيهات الدينية (Religious guidelines) | اللجوء للدعاء، التضرع بالصلاة (Prayer, supplication) | عاجل: هيئة كبار العلماء تدعو على الجميع التقيد بالتعليمات والتوجيهات والتنظيمات وتقوى الله واللجوء إليه بالدعاء والتضرع بالصلاة. كورونا السعودية. |
|  | العمرة (Umrah) | تعليق العمرة (Suspension of Umrah) | اكتشفت في أزمة فيروس كورونا أن حفظ النفس هو أهم مقاصد الشريعة ولها يخضع كل شيء. تم تعليق العمرة وتعليق الصلاة في المساجد مما يدل على عظمة دين الإسلام وعمق مقاصد الشريعة. |
| **Global impact of COVID-19 on sports and countries** | | | |
|  | المباريات (Postponement of matches) | تأجيل المباريات (Postponement of matches) | الاتحاد الأوروبي لكرة القدم يقرر تأجيل جميع مباريات الأسبوع القادم. الرياضة. كورونا. |
|  | ايطاليا (Italy) | وضع ايطاليا (The situation in Italy) | أرقام مرعبة في ايطاليا وايران. فيديو يوضح انتشار فيروس كورونا خارج الصين حتى مارس. |
| **COVID-19 and national efforts** | | | |
|  | الملك سلمان (King Salman) | دعم ملكي (Royal support) | الملك سلمان بن عبدالعزيز وولي العهد الأمير محمد بن سلمان. مؤسسة النقد تعلن دعم القطاع الخاص بمليار ريال لمواجهة الآثار المالية والاقتصادية المتوقعة بسبب فيروس كورونا |
|  | شكر (Thanks) | شكر الحكومة (Government gratitude) | شكراً نحمد الله على نعمة الإسلام ونشكر الله على نعمة سلمان. لكل سعودي يحق له أن يفتخر ويفاخر بالسعودية. حفظ الله حكومتها وشعبها من كل شر. السعودية. كورونا. خليك في البيت. |
